# The Effects of Sit-to-Stand Training Combined with Real-Time Visual Feedback on Strength, Balance, Gait Ability, and Quality of Life in Patients with Stroke: A Randomized Controlled Trial

**DOI:** 10.3390/ijerph182212229

**Published:** 2021-11-21

**Authors:** Seung-Jun Hyun, Jin Lee, Byoung-Hee Lee

**Affiliations:** 1 Graduate School of Physical Therapy, Sahmyook University, Seoul 01795, Korea; asura0486@hanmail.net; 2 Department of Physical Therapy, Sahmyook University, Seoul 01795, Korea; leejin87@hanmail.net

**Keywords:** gait, muscle strength, postural balance, quality of life, real-time feedback, stroke, visual feedback

## Abstract

This study aimed to investigate the effects of lower limbs muscles’ strength, balance, walking, and quality of life through sit-to-stand training combined with real-time visual feedback (RVF-STS group) in patients with stroke and to compare the effects of classic sit-to-stand training (C-STS group). Thirty patients with stroke were randomly divided into two groups. The RVF-STS group received sit-to-stand training combined with real-time visual feedback using a Wii Balance Board (*n* = 15), and the C-STS group received classic sit-to-stand training (*n* = 15). All participants received training for 20 min once a day, 5 days a week for 6 weeks, and both groups underwent general physical therapy for 30 min before training. Before and after the training, the muscle strength of the hip flexor, abductor, and knee extensor were measured, and the Wii Balance Board was used to perform the center of pressure test and Berg Balance Scale to evaluate static and dynamic balance. Additionally, the 10 m walking test and the Timed Up and Go test were performed to evaluate gait function. The Stroke-Specific Quality of Life was used to measure the quality of life. The results showed that the lower extremity muscle strength, balance ability, walking ability, and quality of life of the RVF-STS group significantly improved in comparison of the pre- and post-differences (*p* < 0.05), and it also showed significant differences between groups (*p* < 0.05). This study showed that sit-to-stand training combined with real-time visual feedback was effective at improving the muscle strength of the lower extremities, balance, gait, and quality of life in patients with stroke. Therefore, repeating sit-to-stand training combined with real-time visual feedback could be used as an effective treatment method for patients with stroke.

## 1. Introduction

The severity of stroke depends on the location and extent of the region involved. The most typical symptom is hemiparesis of the body contralateral to the brain lesion, which causes muscle weakness in the upper and lower limbs and limited walking and balance [1]. The sit-to-stand and stand-to-sit positions, normal weight bearing, and walking movements are limited. The movement of the body in daily life is generally affected [2].

The decrease in the balance ability of patients with stroke results in abnormal muscle mobilization on the non-paralyzed side due to the decrease in muscle strength on the paralyzed side, decrease in movement, and difficulty in performing the activities of daily living [3]. These factors increase the risk of falls and cause gait problems [4]. Patients with stroke also experience problems with the static balance in the sitting and standing postures. Moreover, there may also be a problem with dynamic balance, which is experienced during actions such as changing from sit-to-stand and from stand-to-sit positions, resulting in decreased postural stability during static and dynamic standing. They support their weight asymmetrically [5].

Lower extremity muscle strength loss due to neurological damage after a stroke, limited balance, rigidity, and spasticity make normal walking difficult [6]. Over 85% of patients with stroke experience hemiparesis after onset, and 55–75% of survivors experience semi-permanent disorders, such as motor deficits, which can reduce their quality of life [7].

Sit-to-stand motion is one of the functional motor performance abilities that are common in daily life. When standing up, the body weight is supported by the lower extremities, and an efficient standing ability precedes the normal gait. However, patients with stoke tend to fall upon standing due to unstable movement, decreased postural control, and muscle weakness [5]. Additionally, the time from sitting to standing increases [8], and the center of gravity is different in front or from behind and from the right or left [9]. Such sit-to-stand motion requires postural control ability, symmetrical weight bearing of both lower limbs, and lower limb extensor strength [10].

Traditionally, clinical interventions for recovering patients with stroke include Bobath neurodevelopmental treatment [11], proprioceptive neuromuscular palpation [12], and underwater exercise [13]. Additionally, virtual reality training [14] and rehabilitation assistant robot training [15] are widely used. Recent studies have reported that intensive and repetitive training [16], an additional intervention with active participation and motivation [17], visual–motor feedback [18], and auditory–motor feedback [19] are more effective at improving function. In particular, visual feedback can improve the input of impaired and proprioceptive sensory information in patients with stroke and promote walking ability and motor learning [20,21]. A previous study showed that accurate and diverse sensory feedback elicited motivation for active participation and achievement, and it exhibited effects on cognitive, concentration, and motor learning abilities [22].

This study aimed to determine the muscle strength and balancing ability of the lower extremities and their effects on the health, gait, and quality of life of subacute patients with stroke when the patient checks his or her appearance and corrects the compensation effect of the upper body and trunk during the sit-to-stand training through visual feedback. It also aimed to provide information on effective training methods for patients with stroke.

## 2. Materials and Methods

### 2.1. Subjects

The experiment was conducted on 40 patients with subacute stroke who were admitted at hospital B in Seoul. The criteria for selecting the participants were as follows: those diagnosed with hemiparesis due to a stroke between 3 and 6 months after onset [23,24], those who could communicate, understand tasks, and follow directions with more than 21 points in the Mini-Mental State Examination-Korean (MMSE-K) score [25,26,27], those who could independently perform standing motions without using their hands in a sitting position and maintain an independent standing posture for more than 1 min [28,29], those who did not have limb fractures, joint pain, joint motion limitation, and instability and those who could not perform the standing motion [28], and those who did not have amblyopia, hemianopsia, vertigo, and vestibular dysfunction [28].

The present study was approved by the Institutional Review Board of Sahmyook University (Seoul, Korea, 2-7001793-AB-N-012019063HR), and it was registered (KCT0005299) by the Clinical Research Information Service in the Republic of Korea. The objectives and procedures to be performed in the study were fully understood by the participants, and all participants provided informed consent for inclusion in the study. This study was performed according to the ethical principles of the Declaration of Helsinki.

### 2.2. Experimental Procedures

Before recruiting the participants for this study, we performed a power analysis using G*Power version 3.1.9.4 (Heinrich-Heine-Universität, Düsseldorf, Germany, 2019). The overall effect size index for all outcome measures and the power of the study were 0.5, the probability was 0.05, and the type II error (power of 80%) was minimized. As the estimated target sample size was 29, we recruited 40 participants for this study. 

All participants were measured before and after training for 6 weeks using a digitalized manual muscle tester (hand-held dynamometer) and evaluated by the center of gravity test (Center of Pressure test, COP), Berg Balance Scale (BBS), 10 m Walking Test (10MWT), Timed Up and Go Test (TUG), and quality of life scale (Stroke-Specific Quality of Life, SS-QOL). Participants who fulfilled the selection conditions filled a consent form after receiving detailed explanations of the experimental procedure and were randomly classified into two groups: an experimental group trained to sit-to-stand with real-time visual feedback (RVF-STS group, *n* = 20) and a sit-to-stand control (C-STS) group (*n* = 20). In this single-blinded study, all tests were performed and analyzed by the same examiner, and training was performed for each group.

**Figure 1:**
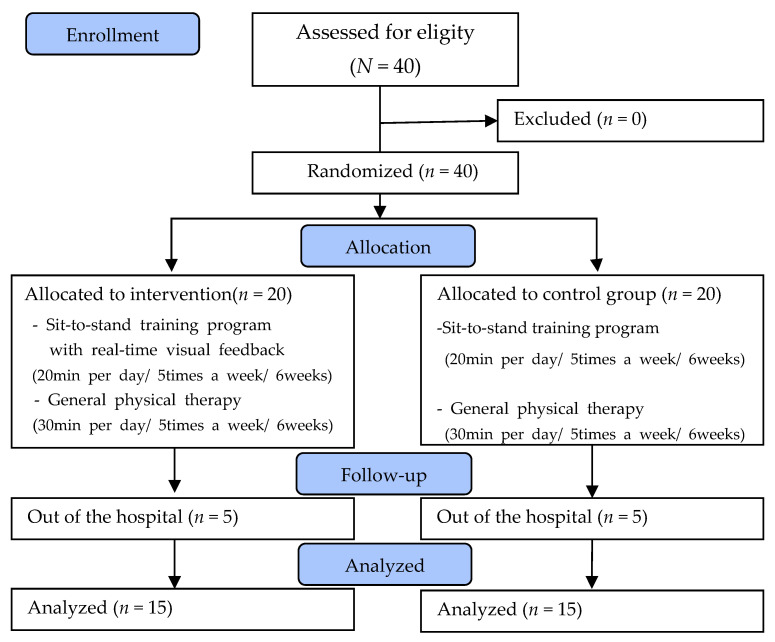
Flow diagram of total experimental procedure.

In both groups, general physical therapy (exercise therapy, electrical stimulation therapy) was performed once a day for 30 min, 5 days a week during the 6-week intervention period. Moreover, the RVF-STS group trained for 20 min once a day for 6 weeks, 5 days a week, and the C-STS group performed the same for 6 weeks. Five patients per group dropped out from the study owing to discharge from the hospital. A total of 30 participants were included in the study (Figure 1). 

#### 2.2.1. The Sit-to-Stand Training Program with Real-Time Visual Feedback

RVF-STS is a motor learning method that uses visual feedback. Real-time feedback is used to control the effects of visual feedback and compensatory movements that can occur during exercise, using the dynamic posturography proposed by Wolfson et al. [30]. The effect of the real-time feedback using a mirror was suggested by Pinches et al. [31]. Based on the combined motor learning method, a sit-to-stand training program with real-time visual feedback was constructed by considering real-time visual feedback and functional improvement.

In this study, the sit-to-stand training method applied to the experimental group was performed by adjusting the height through adding or removing blocks (46 × 69 × 10 cm) to a flat leveling block (leveling block, 46 × 69 × 40 cm) without a back and armrests. The height of the leveling block was adjusted according to each participant’s knee height (height between the lateral epicondylitis of the participants’ femurs from the ground) [32].

The ankle, knee, and hip joints were maintained by flexing to 90°, the distance between the feet was equal to the width of the pelvis, and the distance between the fibula and malleolus was maintained in a parallel position [33]. The depth of the hip supported by the leveling block was half the length of the thigh (from the greater trochanter of the femur to the joint line of the knee) [23]. The participant’s arm was placed comfortably next to his/her body before the training was performed. To prevent compensation when standing up, the paralyzed hand was crossed and supported by the non-paralyzed hand. During the sit-to-stand training, the direction of the gaze was naturally directed to the front. To prevent errors in the study, the positions of both feet and thighs were kept constant using fluorescent colored tape. The definition of a completely standing posture was to completely extend the knee and hip joints after the trunk was erected. The participant attained the maximum standing posture, recognized it, and maintained it during the repetitive standing training [34]. The Wii Balance Board (Nintendo, Kyoto, Japan, 2010) was used as a visual feedback device to provide real-time visual feedback to the participant during the sit-to-stand training. The equipment consisted of two force plates (each 50 × 50 cm) that detected the participant’s weight load, a computer that provided visual feedback (NT900X4C, Samsung, Korea, 2012), a 17-inch monitor (LCD display, IBM, USA, 1993), and a real-time feedback device that used a mirror. The mirror was located just behind the monitor and provided visual feedback to the participant for checking his/her training in real time. Furthermore, fluorescent-colored tape was attached to the center of the mirror, and the face, chest, and abdomen were marked to enable the participant to perform the sit-to-stand motion while maintaining the center as much as possible.

As a method of applying real-time visual feedback, the RVF-STS group looked at the monitor displaying the participant’s center of pressure on two force boards and observed their training in real time through the front mirror. Before the full training, the participants were instructed to get up while maintaining the center of the face, chest, and abdomen as much as possible according to the fluorescent tape marked on the mirror. During the training, the importance of movement was emphasized for the participants to stand up at a stable speed and in a correct posture. The training was discontinued immediately if they experienced fatigue, changes in complexion, or pain.

To prevent falls during the stand-up training, the therapist observed the participants from a supportable distance. The frequency and speed were induced while maintaining a symmetrical posture at a comfortable speed chosen by the participant for 20 min to prevent fatigue [23,28]. The training was conducted for 20 min once a day, 5 days a week for a total of 6 weeks. Each set of 12 sessions was conducted for 20 min with a 1-min rest between sets [35] (Table 1).

#### 2.2.2. Sit-to-Stand Training Program

In this study, the sit-to-stand training method applied to the control group was performed by adjusting the height through adding or removing blocks to a flat leveling block without backrests and armrests. The height of the leveling block was matched to the height of each participant’s knee (the height between the lateral condyle of the participants’ femurs from the ground) [32].

The ankle, knee, and hip joints were maintained by flexing to 90°, and the distance between both feet was equal to the width of the pelvis, and the distance between both calf and ankle bones was maintained and placed in a parallel relation [33]. The depth of the hip supported by the leveling block was half the length of the thigh (from the greater trochanter of the femur to the knee joint line) [23]. The participant’s arm was placed comfortably next to his/her body before the training was performed, and to prevent compensation when standing up, the paralyzed hand was crossed and supported by the non-paralyzed hand. During the sit-to-stand training, the direction of the gaze was naturally directed to the front. To prevent errors in the study, the positions of the feet and thighs were kept constant using fluorescent colored tape. The definition of a completely upright posture was to have the knee joint and hip joint fully extended after the trunk was erected. The participants performed a standing posture as much as possible to stand up, recognize, and maintain it during repetitive standing training [34]. Before the sit-to-stand training, the participant was instructed to stand up while maintaining the center of the face, chest, and abdomen according to the position of the therapist in front. During the training, the importance of movement was emphasized to enable the participants to stand up at a stable speed and in a correct posture. The training was discontinued immediately if they experienced fatigue, changes in complexion, or pain.

To prevent falls during the stand-up training, the therapist observed the participant from a supportable distance. The frequency and speed were induced while maintaining a symmetrical posture at a comfortable speed chosen by the participant for 20 min to prevent fatigue [23,28]. The training was conducted for 20 min once a day, 5 days a week for a total of 6 weeks. Each set of 12 sessions was conducted for 20 min every time with a 1-min rest between sets [35].

#### 2.2.3. General Physical Therapy

General physical therapy was performed using central nervous system development and functional electrical stimulation therapies. The central nervous system development treatment used was the Bobath therapy [36], which is performed for patients in most physical therapy rooms [33]. The general physical therapy for all patients was performed 30 min once a day, 5 days a week for a total of 6 weeks.

### 2.3. Outcome Measurements 

#### 2.3.1. Muscle Strength Test of Lower Extremities

A digitalized manual muscle tester was used to measure the muscle strength of the hip flexors, abductors, and extensors of the knee joint on the paralytic side. The reliability of a digitalized manual muscle tester for measuring lower extremity muscle strength in patients with neurological disorders was reported to be as high as that for the hip flexor (intraclass correlation coefficient (ICC) = 0.86), hip abductor (ICC = 0.76), and knee joint extensor (ICC = 0.83) [37]. To measure the strength of the hip flexor muscles, the participant sat in a chair with back support with the hip and knee joints flexed at 90° and the ankle joint in a neutral position. To measure the strength of the hip flexor, the participant sat in a chair with back support, flexing the hip and knee joints at 90°, and the ankle joint in a neutral position. The location of the measuring instrument was assessed at the end of the upper femur. To measure the strength of the hip abductor, the participant laid in a side-lying position; the hip and knee joints were extended while their feet maintained contact with the floor. To eliminate the compensation, the examiner kneeled to the back and fixed the pelvis of the paralyzed side using a single hand. The measuring device was placed at the midpoint of the anterior superior iliac spine of the lateral femur and patella. To measure the strength of the knee joint extensors, the participant sat in a chair with a back support, with the hip and knee joints flexed at 90°, and the ankle joint in a neutral position. The measuring device was on the ventral surface of the tibia and 5 cm proximal to the inferior medial malleolus. Before the measurement, the participants practiced twice to train themselves for the test method, with 2 min of rest provided for each measurement to minimize muscle fatigue. At the beginning of the measurement, the participants were instructed to push against the resistance with the greatest force possible. All measurements were obtained two times, and after three measurements, the average value was recorded [38].

#### 2.3.2. Balance

The static and dynamic balance abilities were measured. To evaluate the participant’s static balance ability, the COP test was performed using a Wii Balance Board (Nintendo, Kyoto, Japan, 2010). The Balancia program (Balancia software ver. 2.0, Mintosys, Seoul, Korea) was used to analyze the data. The pressure center movement test measured the body pressure center movement information (COP path length) on the *X*- and *Y*-axis applied to the force plate supporting two feet and is an index indicating the balance of the standing posture and the stability of the center of gravity. The smaller the value, the smaller the fluctuation of the pressure center. The inter-measure reliability for the pressure center movement test of the Balancia program was ICC = 0.79–0.96, and the validity was ICC = 0.85–0.96 [39]. The participant stood on the strength plate, with a forward gaze and both arms comfortably placed next to the trunk. The test was conducted by standing on the strength plate for 30 s with their eyes open. Changes in the center of body pressure applied to the force plate were tracked using a monitor, and the values were measured based on the width and height of the shaking. The test was repeated if the balance was lost or the foot deviated from the force plate.

To evaluate the dynamic balance ability, the BBS, a test tool for the balance between elderly and nervous system disorder patients at high risk of falls, was used. It comprises a 5-point scale (0–4) composed of 14 items with a total score of 56 [40]. In the BBS, the intra-measure reliability was ICC = 0.98, and the inter-measure reliability was ICC = 0.97, showing high reliability and internal validity [41]. In general, participants with scores of 0–20 points use wheelchairs, 21–40 points require an assist device or assistance, and 41 points or more have independent gait [42].

#### 2.3.3. Gait

In this study, TUG and 10MWT were used to measure the walking ability. TUG is a test that can measure functional mobility, mobility, and balance in a short time. After sitting in a chair with an armrest height of 46 cm, it measured the time from standing up with the experimenter’s starting signal, walking a distance of 3 m, returning, and sitting back on the chair. The intra-measure reliability of this test was reported as ICC = 0.99, and the inter-measure reliability was ICC = 0.98 [43]. All tests were repeated thrice to obtain an average value.

The 10MWT is a test to evaluate the gait performance and is measured as a self-selective gait speed that allows the participant to walk while feeling safe [44]. It sets 2 m at the beginning and end as the distance for acceleration and deceleration along a 14 m linear distance. It also measures the walking time for a distance of 10 m, excluding the 2 m at the beginning and end [45]. The intra- and inter-measure reliability of this test was reported as ICC = 0.89–1.00 [46]. All tests were repeated thrice to obtain an average value.

#### 2.3.4. Quality of Life

In this study, the SS-QOL, developed by Williams et al. [47], was used to measure the quality of life of the participant. The SS-QOL is a scale for patients with stroke. There are 12 domains: energy (three items), family role (three items), language use (five items), movement (six items), mood (five items), personal personality (three items), self-help activities (five items), social role (five items), thinking ability (three items), upper limb function (five items), vision (three items), and occupation-production activities (three items). It is a 5-point scale consisting of 49 items, with the lowest score of 49 and highest score of 245. The lower the score, the higher the quality of life. The reliability of this test was reported as a Cronbach’s α ≥ 0.73 [47]. In this study, the evaluation tool modified, supplemented, and suggested by Moon [48] was used, and the reliability was a Cronbach’s α ≥ 0.80.

### 2.4. Statistical Analysis

Statistical analysis was performed using the SPSS statistical software (IBM, Chicago, IL, USA), version 21.0. The Shapiro–Wilk test was used to analyze the normal distribution of the general characteristics and variables. The independent *t*-test was used to compare the homogeneity between the groups. The paired *t*-test was used to compare the results pre- and post-intervention. Lastly, the analysis of covariance was performed to identify differences between the groups. The level of statistical significance was set at *p* < 0.05. 

## 3. Results

The demographic characteristics of the participants are shown in Table 2. No significant differences were observed in the baseline values between the RVF-STS and control groups for all parameters.

### 3.1. Muscle Strength of the Lower Extremities

Covariance analysis was conducted to determine whether there was a difference between the groups in terms of the level of change in the lower extremity muscle strength after training. The lower extremity muscle strength was processed as a covariate (Table 3). The results of the study showed statistically significant differences in the muscle strength of the hip flexor (F = 6.690; *p* < 0.015), hip abductor (F = 6.930; *p* < 0.014), and knee extensor (F = 6.152; *p* < 0.02). These results indicate that the RVF-STS group had a statistically significant improvement in the lower extremity muscle strength compared to that in the C-STS group, demonstrating that the training program in this study was effective.

### 3.2. Balance

Covariance analysis was conducted to determine whether there was a difference between the groups in terms of the level of change in balance after training. The COP and BBS was processed as a covariate (Table 4). The results showed statistically significant differences in COP (F = 10.849; *p* < 0.003), and BBS (F = 5.403; *p* < 0.028). These results indicate that the RVF-STS group had a statistically significant improvement in balance compared to that in the C-STS group, demonstrating that the training program in this study was effective.

### 3.3. Gait

Covariance analysis was conducted to determine whether there was a difference between groups in the change in gait after training. The sit-to-stand test and 10 m walk test was processed as a covariate (Table 5). The results showed statistically significant differences in TUG (F = 7.207; *p* < 0.012) and 10MWT (F = 5.796; *p* < 0.023). These results indicate that the RVF-STS group had a statistically significant improvement in gait compared to that in the C-STS group, demonstrating that the training program in this study was effective.

### 3.4. Quality of Life 

Covariance analysis was conducted to determine whether there was a difference between groups in the change in quality of life after training. The SS-QOL was processed as a covariate (Table 6). The results showed statistically significant differences in SS-QOL (F = 28.050; *p* < 0.000) and 10 m walk test (F = 5.796; *p* < 0.023). These results indicate that the RVF-STS group had a statistically significant improvement in the quality of life compared to that in the C-STS group, demonstrating that the training program in this study was effective.

## 4. Discussion

The weakening of muscle strength in patients with stroke is an important factor that decreases the gait speed. There is a positive correlation between the muscle strength and maximum gait speed [49]. In particular, the hip flexors have a significant correlation with the gait speed, and the knee joint extensors have a high correlation with the gait stability [50]. Hip abductors are important for restoring balance and independent gait. Moreover, since it stabilizes the hip joint posture in the standing phase [51], strength training is critical, and the effect of increasing the muscle strength is particularly high in early patients with stroke [52]. In the RVF-STS group, the muscle strength of the hip flexors, hip abductors, and knee extensors increased from 9.66 to 12.46 kg, 9.44 to 11.66 kg, and 14.66 to 19.96 kg, respectively. There was a significant improvement in both groups (*p* < 0.05). In comparing the difference between the two groups, the RVF-STS group showed a significant difference in the results when compared with the C-STS group (*p* < 0.05).

In this study, the improvement of the lower extremity muscle strength was the maximum activity of the quadriceps muscle, while the directionality of the sit-to-stand motion shifted from the front to the vertical direction. As the hip and knee joints remain active until they are extended [53], the strength of the knee extension improved as the direction of the knee joint moved from a low to a high position during the sit-to-stand training. Additionally, in the initial 40% of the sit-to-stand motions, the hip flexors were mainly activated, the maximum hip flexor angle was 116.8°, the knee extensor strength was activated to the maximum value [54], and the hip flexion strength increased when the hip joint was flexed. Mechanically, the hip abductor stabilizes from the front and the sides to support the body weight during gait [55]. Visual feedback training using a mirror for patients with stroke is a method of inducing a patient’s concentration and repetitive learning by increasing the weight-bearing of the lower extremities using visual information [56]. Through this method, the asymmetry of the body was continuously corrected during the sit-to-stand training, and accurate movements were repeatedly performed while assigning weight to the paralyzed lower limb to enable bilateral exercise training and enhance the strength of the lower limb. Furthermore, it induces precise movements through visual targets in real time and improves motivation and concentration, thus minimizing the compensation of other muscles [57]. In this study, weight was gradually shifted to the paralyzed lower extremity, and the exercise was performed intensively in the correct posture by minimizing the compensation effect to improve the strength of the lower limbs. Balance ability is the ability to maintain the center of gravity on top of one’s base of support in various environments and tasks in daily life [58]. 

In this study, the shift of the pressure center of the RVF-STS group decreased from 94.11 to 72.93 cm, there were significant differences in both the C-STS and RVF-STS groups (*p* < 0.05), and there was a significant difference in the results between the experimental group and the control group (*p* < 0.05).

The improvement of balance ability changes the base surface consisting of three points to two points during the stand-up motion. Since the base surface is reduced, it is possible to increase the ability to adjust the posture along with the change in balance. It causes postural fluctuations in the anterior and posterior directions, and as the balance ability improves, the extensors of the lower extremities biomechanically accelerate the body vertically to maintain the balance in a standing position with a high body center point during the sit-to-stand motion [59]. The strengthening of the knee extensor and improvement of the center of pressure affects the body symmetry, thus improving the static balance [60]. Therefore, RVF-STS improved the knee joint extensors and movement of the center of pressure, thereby improving the static balance. Additionally, the central nervous system responds and predicts in advance through the visual, vestibular, and somatosensory systems to adjust the posture fluctuation and balance [61]. Visual feedback plays an important role in the integration of somatosensory and visual information during voluntary movement, and it can also improve the symmetry and functional movement of the body [62]. Feedback training using vision is stimulated by sight, vestibular sensation, and somatosensory sensation or causative information performed by oneself; it corrects errors in task performance and induces learning of correct movements [63]. Therefore, the body asymmetry was continuously corrected through visual cues that aligned the body centerline during the sit-to-stand training through real-time visual feedback. It corrects errors in weight shift to the paralyzed side and repeat movements for accurate weight shift. In the paralyzed lower extremity, while looking directly at the state of balance and movement, along with a decrease in sway in the paralyzed lower limb and the action of real-time visual feedback that can control balance, the mechanism of control of the biofeedback and bio-feedforward is improved by enhancing the ability to integrate the movement and the senses, thereby enhancing balance.

Along with balance ability, gait ability is evaluated as the most important function in hemiplegic patients due to stroke. The gait speed of patients with stroke decreases by 17–49% compared to that in normal adults [64]. Therefore, gait rehabilitation of patients with stroke is an important goal to improve the function of activity [65]. In this study, the TUG of the experimental group decreased from 20.70 to 16.69 s. There was a significant difference in both groups. Regarding the difference between the two methods of training, the RVF-STS group showed a significant difference in results when compared with the C-STS group (*p* < 0.05). Additionally, the 10 m walk of the experimental group decreased from 26.05 to 11.42 s. There was a significant difference in both the experimental and control groups (*p* < 0.05). Between the two methods of training, the RVF-STS group showed a significant difference in the results compared to the C-STS group (*p* < 0.05).

The improvement in gait ability has been reported owing to the influence of the hip and knee joint extensors during the middle stance phase, which determines the stride rate and step length of the opposite leg, resulting in a difference in gait speed [66]. The improvement of weight support and stability of the paralyzed leg affect gait speed and endurance [67]. Lotte et al. [68] stated that functional movements incorporating ten exercise programs increased the gait speed as well as the balance ability and coordinated movements between the trunk and limbs by improving the postural control ability [69]. Visual feedback aims to improve the motor performance by promoting motor learning. Posture training using visual feedback was more effective in increasing the weight-bearing time on the paralyzed side and improving the gait pattern and speed than was the traditional stand-up balance training method [70]. Additionally, performing an exercise using visual feedback enables self-correction through continuous visual information and can lead to positive nerve recovery through repetitive stimulation [71]. Body symmetry can be restored through real-time visual information, and functional movement can also be improved. By promoting motor and somatosensory sensation, it adjusts the left and right body symmetry, thereby improving the truncal control ability to improve the balance and walking function [72]. Gait is a regulated activity of a network that includes the cortical, subcortical, and spinal regions [73]. Treatment with a mirror stabilizes the activity of the primary motor cortex, which is a key area in the development of paralysis and convulsions; it also promotes nerve mediation and consequently restores motor command execution and function [74]. Therefore, through RVF-STS, weight transfer to the paralyzed side was improved during the normal sit-to-stand movement. When standing up, the knee extensors were activated to increase the experience and time, as in the middle stance phase. By utilizing real-time visual feedback using a mirror, the left–right symmetry was improved by the coordinated movement of the trunk and limbs, thereby improving the stability and posture control of the trunk. Moreover, the gait speed was improved by promoting the reorganization of neural circuits in the cerebral cortex through the feedback mechanism.

After a stroke, patients experience difficulties in independent daily activities, and their quality of life deteriorates due to life changes [75]. In this study, the effect on the quality of life of subacute patients with stroke was investigated through the results of the SS-QOL scale according to whether real-time visual feedback was used in the sit-to-stand training. The quality of life of the experimental group decreased from 149.93 to 116.60 points, and there were significant differences in both groups (*p* < 0.05). The RVF-STS group showed a significant difference compared to the C-STS group (*p* < 0.05). Through visual feedback, the therapist and patient may attempt numerous processes for planning the exercise pattern to achieve the goal and providing accurate knowledge of the patient’s effort to help in rehabilitation [76]. The sub-items of the SS-QOL include mobility, language, vision, thinking, upper extremity function, self-care, energy, social role, personality, work/productivity, and mood. In patients with stroke, there are various factors affecting quality of life, such as physical, psychological, social, and environmental aspects, especially social and physical domains that affect the period of rehabilitation [77,78]. Therefore, the motivation of patients with stroke in this study was evaluated by determining their participation and achievement through the score differences in SS-QOL. 

Social participation is considered a major relevant and pivotal outcome indicating a successful recovery in patients with stroke [79]. The International Classification of Functioning, Disability, and Health defines participation as “involvement in a life situation” or as “the lived experience” of people in the actual context in which they live [80]. Social participation is one of the most important end points of recovery from a stroke and is the goal of rehabilitation [81]. Exercise can improve mobility, balance, fatigue, and endurance after stroke, which may enable the stroke survivor to engage in activities [82,83]. Physical activity is also known to improve a few the secondary effects of stroke, which may be a barrier to social participation [81]. This study is considered to have provided a form of social participation, as training for patients with stroke was conducted in hospitals, which were different from their homes. Finally, the results of this study may increase the general knowledge on participation in an area beyond the sit-to-stand training combined with real-time visual feedback, which may enable therapists to provide more holistic treatment for clients after a stroke.

## 5. Conclusions

This study was conducted to investigate the effect of sit-to-stand training with real-time visual feedback on the lower extremity muscle strength, balance, gait, and quality of life in patients with subacute stroke. The results showed that the RVF-STS significantly improved the lower extremity muscle strength, balance, gait, and quality of life of the participants compared to that observed with the sit-to-stand training (*p* < 0.05). It was demonstrated that RVF-STS had a greater effect on these parameters than did the sit-to-stand training alone. Therefore, as a therapeutic approach in future clinical trials to improve these parameters in subacute patients with stroke, we suggest that RVF-STS is an effective intervention method.

## Figures and Tables

**Table 1:** Sit-to-stand training program.

RVF-STS Program	Sit-to-Stand Training Program	Times
Looked at the monitor displaying the participant’s center of pressure on two force boards and observed their training in real-time through the front mirror during sit-to-stand training.	General sit-to-stand training	12 times per set, 20 min once a day for 6 weeks

RVF-STS = Real-Time Visual Feedback Sit-to-Stand Training. The total exercise time is 20 min, and the rest time between sets is 1 min.

**Table 2:** General characteristics of subjects (*N* = 30).

Parameters	RVF-STS Group (*n* = 15)	Control Group (*n* = 15)	*t(p)*
Gender (male/female)	6/9	7/8	0.357(0.724)
Age (year)	61.47(11.08)	59.27(17.00)	0.412(0.684)
Height (cm)	160.53(9.53)	163.40(9.38)	−0.830(0.413)
Weight (kg)	61.50(9.73)	64.05(17.77)	−0.488(0.629)
Cause (infarction/hemorrhage)	11/4	10/5	−0.386(0.702)
(right/left)	9/6	7/8	−0.714(0.481)
(month)	4.93(1.62)	4.60(1.60)	0.567(0.575)
MMSE-K (score)	26.73(2.12)	26.07(1.98)	0.890(0.381)
MAS (score)	0.20(0.41)	0.13(0.35)	0.475(0.638)
Brunnstrome recovery stage (stage)	4.20(0.68)	4.40(0.63)	−0.837(0.410)

Values are expressed as mean (standard deviation); RVF-STS, Real-Time Visual Feedback Sit-to-Stand Training; MMSE-K, Mini-Mental State Examination-Korean; MAS, Modified Ashworth Scale.

**Table 3:** Comparison of muscle strength of the lower extremities (*N* = 30).

Parameters		Pre-Test	Post-Test	SS	df	MS	*t*(*p*)/F(*p*)
hip flexor (kg)	RVF-STS	9.66(1.92) ^a^	12.46(3.07)				−6.017(0.000)
	Control	9.87(1.83)	11.17(3.s25)				−2.655(0.019)
	Covariate			195.34	1	195.34	
	Group			19.14	1	19.14	6.690(0.015)
	Error			77.25	27	2.86	
hip extensor (kg)	RVF-STS	9.44(1.44)	11.66(2.62)				−5.503(0.000)
	Control	9.48(2.00)	10.32(2.76)				−2.178(0.047)
	Covariate			145.33	1	145.33	
	Group			14.48	1	14.48	6.930(0.014)
	Error			56.43	27	2.09	
Knee extensor (kg)	RVF-STS	14.86(1.16)	19.96(2.60)				−7.650(0.000)
	Control	15.75(1.48)	17.82(3.50)				−2.495(0.026)
	Covariate			10.95	1	10.95	
	Group			53.80	1	53.80	6.152(0.020)
	Error			236.11	27	8.74	

^a^ Mean (Standard Deviation); RVF-STS, Real-Time Visual Feedback Sit-to-Stand Training Group; SS, Sum of Squares; MS, Mean Square.

**Table 4:** Comparison of balance (*N* = 30).

Parameters		Pre-Test	Post-Test	SS	df	MS	*t*(*p*)/F(*p*)
COP (cm)	RVF-STS	94.11(11.31) ^a^	72.93(8.17)				9.413(0.000)
	Control	95.99(10.84)	82.10(10.90)				8.640(0.000)
	Covariate			1583.03	1	1583.03	
	Group			471.68	1	471.68	10.849(0.003)
	Error			1173.88	27	43.47	
BBS (score)	RVF-STS	37.20(10.00)	51.27(5.56)				−5.503(0.000)
	Control	41.60(8.90)	47.73(8.62)				−2.178(0.047)
	Covariate			333.48	1	333.48	
	Group			205.76	1	205.76	6.930(0.014)
	Error			1028.24	27	38.08	

^a^ Mean (Standard Deviation); RVF-STS, Real-Time Visual Feedback Sit-to-Stand Training Group; SS, Sum of Squares; MS, Mean Square; COP, Center of Pressure; BBS, Berg Balance Scale.

**Table 5:** Comparison of gait (*N* = 30).

Parameters		Pre-Test	Post-Test	SS	df	MS	*t*(*p*)/F(*p*)
TUG (sec)	RVF-STS	20.70(9.15) ^a^	16.69(7.91)				5.525(0.000)
	Control	20.85(7.83)	19.27(7.85)				2.447(0.028)
	covariate			1575.26	1	1575.26	
	Group			45.03	1	45.03	7.207(0.012)
	Error			168.72	27	6.24	
10MWT (sec)	RVF-STS	26.05(13.12)	11.42(2.91)				4.718(0.000)
	Control	20.24(12.00)	15.14(9.37)				2.165(0.048)
	covariate			274.48	1	274.48	
	Group			207.83	1	207.83	5.796(0.023)
	Error			968.12	27	35.85	

^a^ Mean (Standard Deviation); RVF-STS, Real-Time Visual Feedback Sit-to-Stand Training Group; SS, Sum of Squares; MS, Mean Square; TUG, Timed Up and Go; 10MWT, 10 Meter Walk Test.

**Table 6:** Comparison of quality of life (*N* = 30).

Parameters		Pre-Test	Post-Test	SS	df	MS	*t*(*p*)/F(*p*)
SS-QOL (score)	RVF-STS	149.93(23.28) ^a^	116.60(16.78)				12.732(0.000)
	Control	164.87(23.96)	144.07(23.96)				8.020(0.000)
	Covariate			14,086.24	1	14,086.24	
	Group			1809.08	1	1809.08	28.050(0.000)
	Error			1741.34	27	64.49	

^a^ Mean (Standard Deviation); RVF-STS, Real-Time Visual Feedback Sit-to-Stand Training Group; SS, Sum of Squares; MS, Mean Square; SS-QOL, Stroke-Specific Quality of Life.

## Data Availability

Not applicable.
